# Author Correction: Iron sulfide-catalyzed gaseous CO_2_ reduction and prebiotic carbon fixation in terrestrial hot springs

**DOI:** 10.1038/s41467-024-55040-0

**Published:** 2024-12-06

**Authors:** Jingbo Nan, Shunqin Luo, Quoc Phuong Tran, Albert C. Fahrenbach, Wen-Ning Lu, Yingjie Hu, Zongjun Yin, Jinhua Ye, Martin J. Van Kranendonk

**Affiliations:** 1 State Key Laboratory of Palaeobiology and Stratigraphy, Nanjing Institute of Geology and Palaeontology, Chinese Academy of Sciences, 210008 Nanjing, China; 2 International Center for Materials Nanoarchitectonics (WPI-MANA), National Institute for Materials Science (NIMS), 1-1 Namiki, Tsukuba, Ibaraki 305-0044 Japan; 3 School of Chemistry, University of New South Wales, Sydney, NSW 2052 Australia; 4 Australian Centre for Astrobiology, University of New South Wales, Sydney, NSW 2052 Australia; 5 UNSW RNA Institute, University of New South Wales, Sydney, NSW 2052 Australia; 6 National Key Laboratory of Uranium Resource Exploration-Mining and Nuclear Remote Sensing, East China University of Technology, 330013 Nanchang, China; 7 State Key Laboratory of Nuclear Resources and Environment, East China University of Technology, 330013 Nanchang, China; 8 Nanjing Key Laboratory of Advanced Functional Materials, Nanjing Xiaozhuang University, 211171 Nanjing, China; 9 Graduate School of Chemical Sciences and Engineering, Hokkaido University, Sapporo, Hokkaido 060-0814 Japan; 10 TJU-NIMS International Collaboration Laboratory, School of Materials Science and Engineering, Tianjin University, 300072 Tianjin, China; 11 School of Biological, Earth, and Environmental Sciences, University of New South Wales, Sydney, NSW 2052 Australia; 12 School of Earth and Planetary Sciences, Curtin University, Bentley, 6845 Western Australia

**Keywords:** Astrobiology, Geochemistry

Correction to: *Nature Communications* 10.1038/s41467-024-54062-y, published online 28 November 2024

In this article the affiliation details for Jingbo Nan were incorrectly given as ‘Nanjing Institute of Geology and Palaeontology, Chinese Academy of Sciences, 210008 Nanjing, China’ but should have been ‘State Key Laboratory of Palaeobiology and Stratigraphy, Nanjing Institute of Geology and Palaeontology, Chinese Academy of Sciences, 210008 Nanjing, China.’. The original article has been corrected.

