# Supplementary material for: Iron sulfide-catalyzed gaseous CO2 reduction and prebiotic carbon fixation in terrestrial hot springs
Source: Nat Commun. 2024 Nov 28;15:10280. doi: 10.1038/s41467-024-54062-y (PMC11605115; doi:10.1038/s41467-024-54062-y)
Supplement: Supplementary file 1 — Supplementary Information [file 41467_2024_54062_MOESM1_ESM.pdf]

Iron sulfide-catalyzed gaseous CO_2 reduction and prebiotic carbon fixation in terrestrial hot springs

Jingbo Nan[1,#], Shunqin Luo[2,#,*], Quoc Phuong Tran[3,4], Albert C. Fahrenbach[3,4,5], Wen-Ning Lu[6,7,2], Yingjie Hu[8], Zongjun Yin[1], Jinhua Ye[2,9,10,*], Martin J. Van Kranendonk[4,11]

[1]Nanjing Institute of Geology and Palaeontology, Chinese Academy of Sciences, Nanjing 210008, China

[2]International Center for Materials Nanoarchitectonics (WPI-MANA), National Institute for Materials Science (NIMS), 1-1 Namiki, Tsukuba, Ibaraki 305-0044, Japan

[3]School of Chemistry, University of New South Wales, Sydney, NSW 2052, Australia

[4]Australian Centre for Astrobiology, University of New South Wales, NSW 2052, Australia

[5]UNSW RNA Institute, University of New South Wales, Sydney, NSW 2052, Australia

[6]National Key Laboratory of Uranium Resource Exploration-Mining and Nuclear Remote Sensing, East China University of Technology, Nanchang 330013, China

[7]State Key Laboratory of Nuclear Resources and Environment, East China University of Technology, Nanchang, 330013, China

[8]Nanjing Key Laboratory of Advanced Functional Materials, Nanjing Xiaozhuang University, Nanjing 211171, China

[9]Graduate School of Chemical Sciences and Engineering, Hokkaido University, Sapporo, Hokkaido 060-0814, Japan

[10]TJU-NIMS International Collaboration Laboratory, School of Materials Science and Engineering, Tianjin University, Tianjin 300072, China

[11]School of Biological, Earth, and Environmental Sciences, University of New South Wales, NSW 2052, Australia

[#]These authors contributed equally: Jingbo Nan, Shunqin Luo

*Corresponding authors: Shunqin Luo, Jinhua Ye

Email: luoshunqin1116@163.com, jinhua.ye@nims.go.jp

Supplementary information

1) Additional Characterizations of Pure FeS and Mn-Doped FeS
2) Experimental Apparatus
3) H_2-Dependent CO_2 Reduction by Pure FeS and FeS Doped with Different Hot Spring Metals (Ni, Co, and Ti)
4) H_2-Dependent CO_2 Reduction by Mn-Doped FeS Under Simulated Hot Spring Conditions
5) Parameters Used in the DFT Calculations
6) SI Methods

1. Additional Characterizations of Pure FeS and Mn-doped FeS

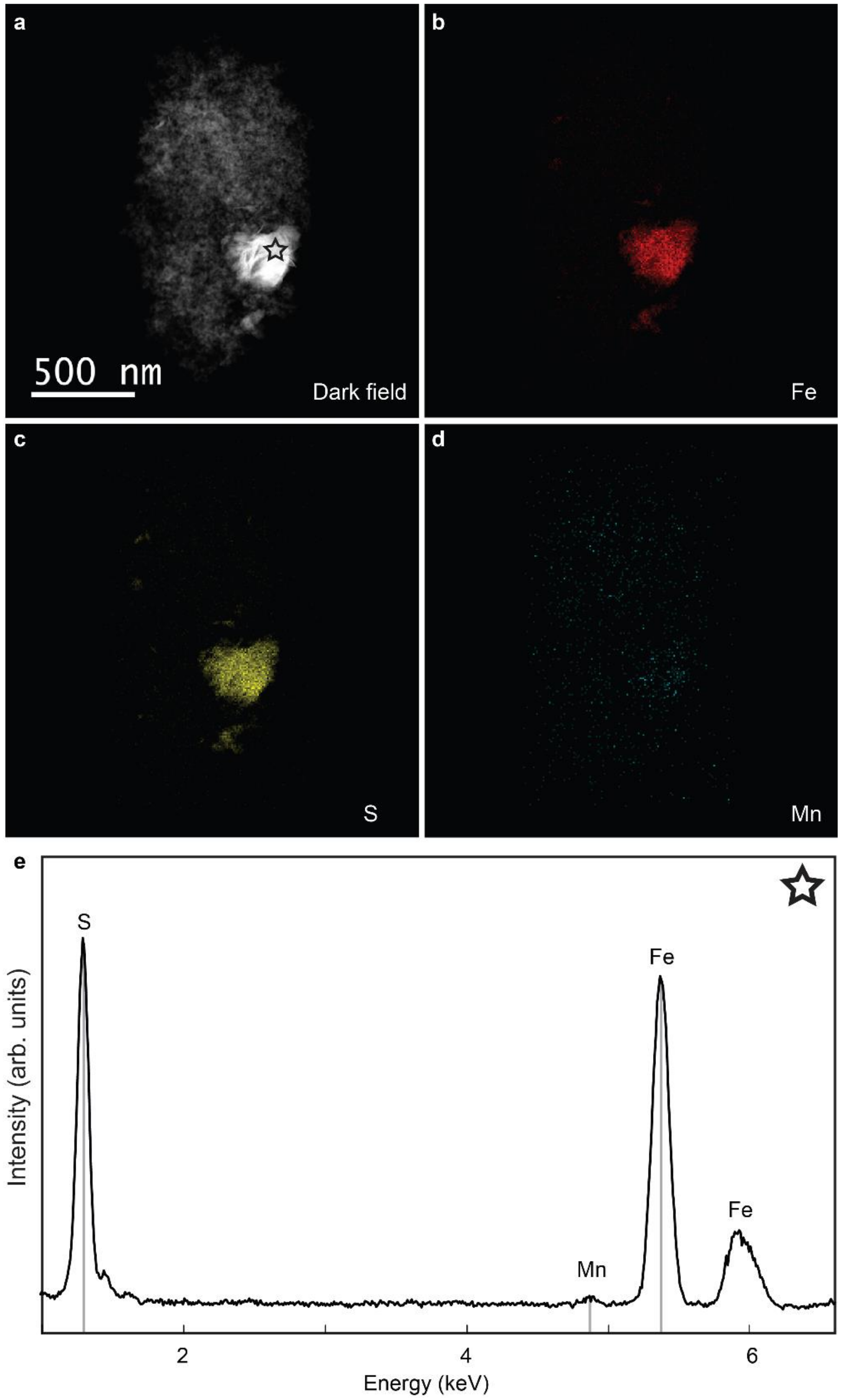

Figure S1. Characterization of the Mn-doped FeS catalyst. **a** Dark-field scanning transmission electron microscopy (STEM) image showing irregular or plate-like crystalline structure of the resultant FeS nanoparticles. **b–d** Corresponding energy-dispersive X-ray (EDX) spectroscopy maps in **a** for iron (red), sulfur (yellow), and manganese (blue). **e** EDX spot analysis of the "starred" location in **a**.

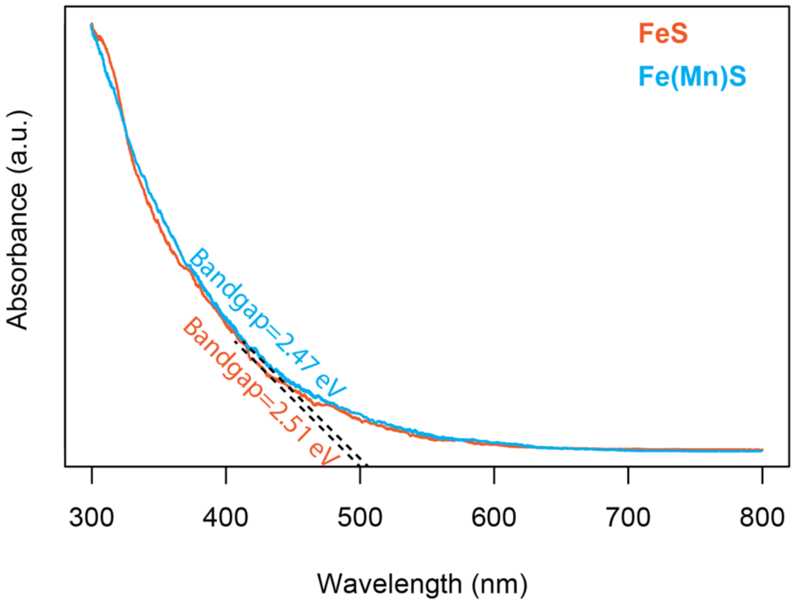

Figure S2. Ultraviolet–visible diffuse reflectance spectroscopy analysis of pure FeS and Mn-doped FeS. The overlap between the two spectra signifies the similar light absorption and electronic structures of pure FeS and Mn-doped FeS.

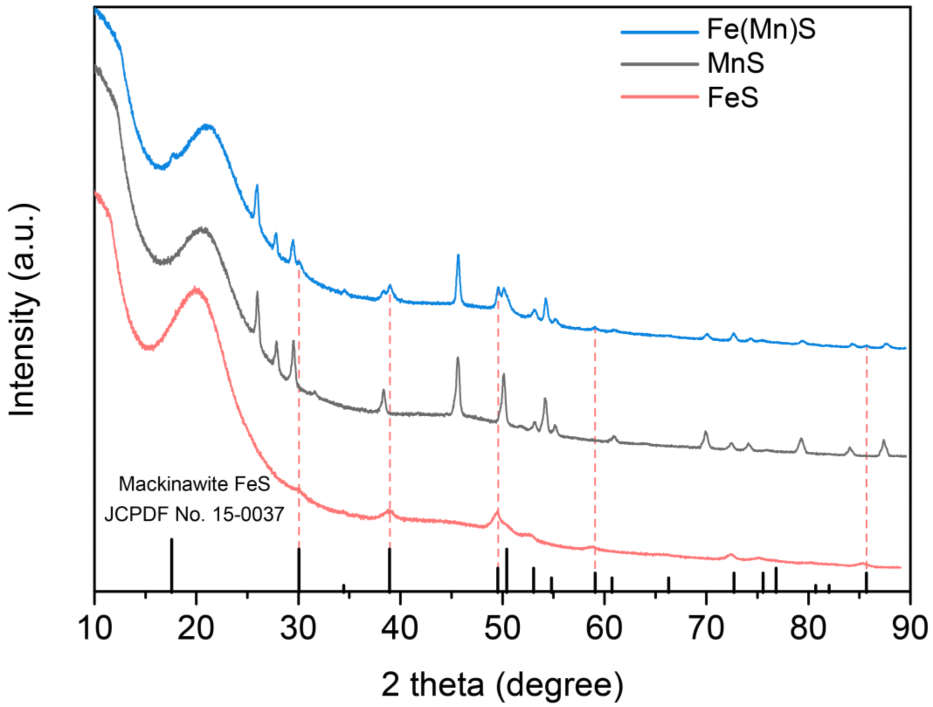

Figure S3. X-ray diffraction (XRD) patterns for FeS, MnS, and Fe(Mn)S (doping ratio at 10%) catalysts. The reference peaks for mackinawite FeS (JCPDF No. 15-0037) are marked with vertical dashed lines, highlighting the presence of Mn-doped FeS in the mixture, although MnS is also present.

Table S1. Surface area and crystalline size of FeS and Fe(Mn)S catalysts.

Sample name	Specific surface area ($m^2\ g^{-1}$)[a]	Crystalline size (nm)[b]
FeS	167.8	12.2
Fe(Mn)S	166.4	13.0

[a] analyzed by the Brunauer-Emmett-Teller (BET) method on the basis of nitrogen adsorption/desorption isotherms

[b] estimated by the Scherrer equation based on the X-ray diffraction (XRD) analysis

2. Experimental Apparatus

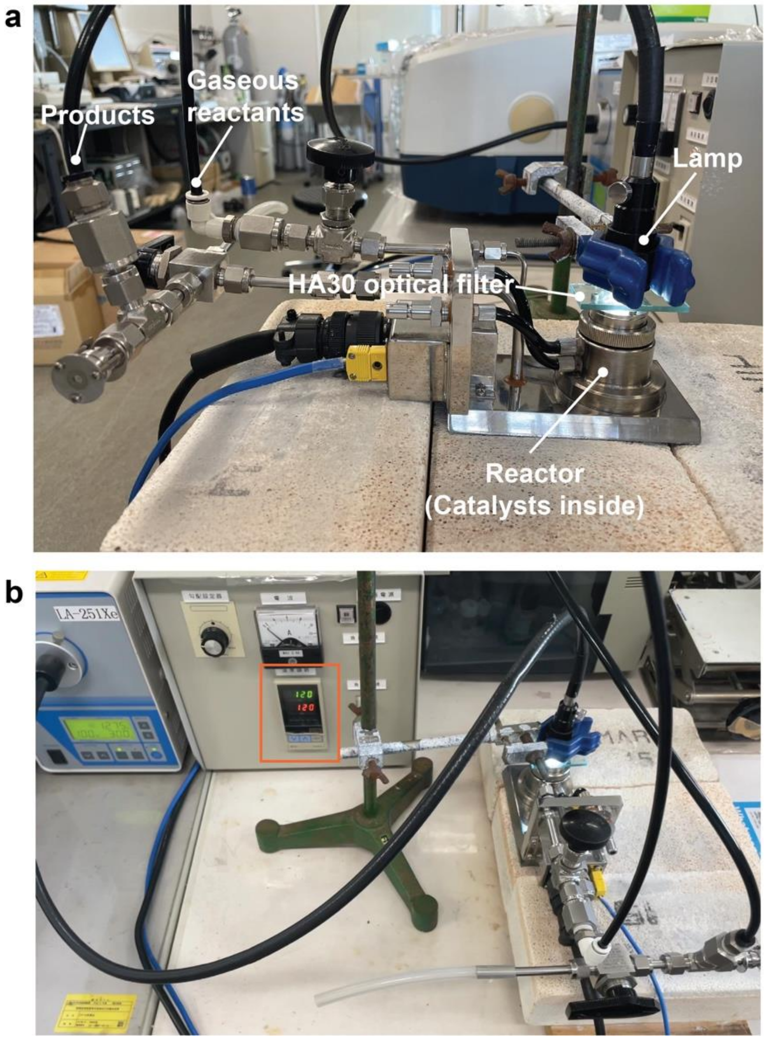

Figure S4. Customized setup for photo-assisted thermocatalysis experiments. **a** The image of the reactor configured for photo-assisted thermocatalytic reactions, featuring a quartz window with HA30 optical filter that permits light to penetrate during the thermocatalytic process while blocking infrared light to minimize photothermal effects. A resistive heater, governed by a temperature controller, is employed to maintain the catalyst at the set temperature, countering any photothermal heating. **b** The catalyst temperature during photo-assisted thermocatalytic reactions was monitored. The temperature controller, coupled with a thermocouple (TC-1000, JASCO), precisely heated the reaction bed, ensuring that the catalysts remained at the targeted temperatures and compensating for any additional heat from light exposure.

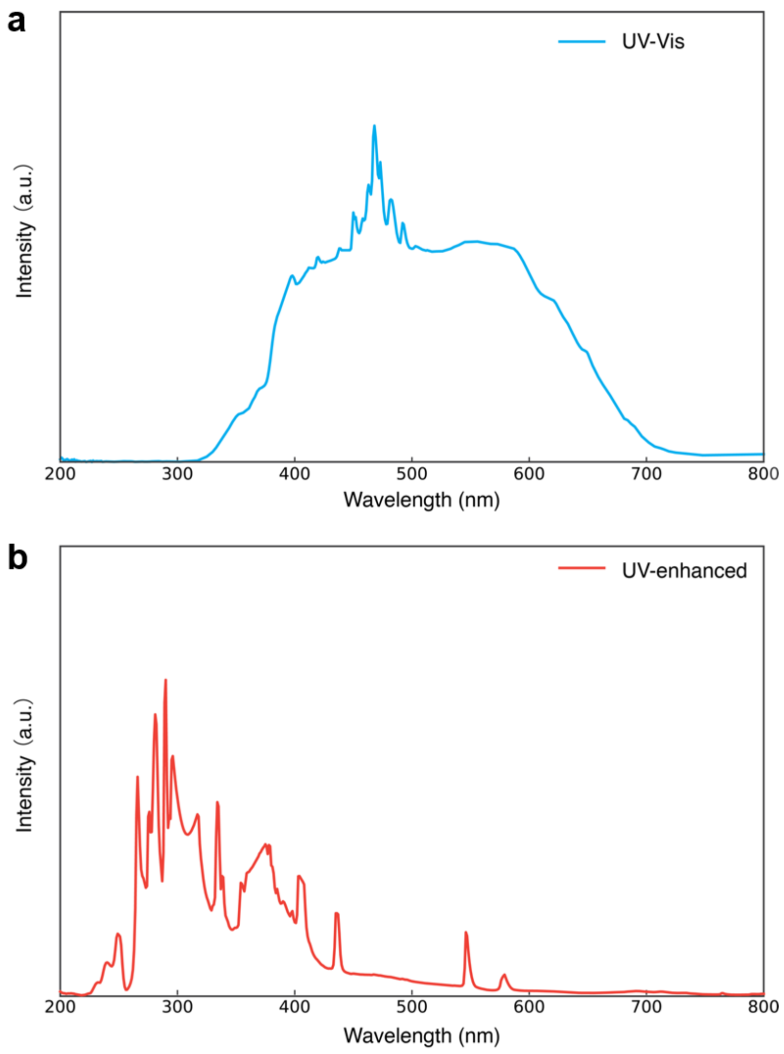

Figure S5. Output irradiance of UV–visible light and UV-enhanced light. **a** Emission of the broad-range UV–visible light (300 < λ < 720 nm). LA-251 Xe lamp with an HA30 optical filter was used to provide UV–visible light irradiation. **b** Emission of UV-enhanced light (200 < λ < 600 nm). LC8 L9566-01A light (Hamamatsu) lamp was used without any optical filter to provide UV-enhanced light.

3. H_2-Dependent CO_2 Reduction by Pure FeS and FeS Doped with Different Hot Spring Metals

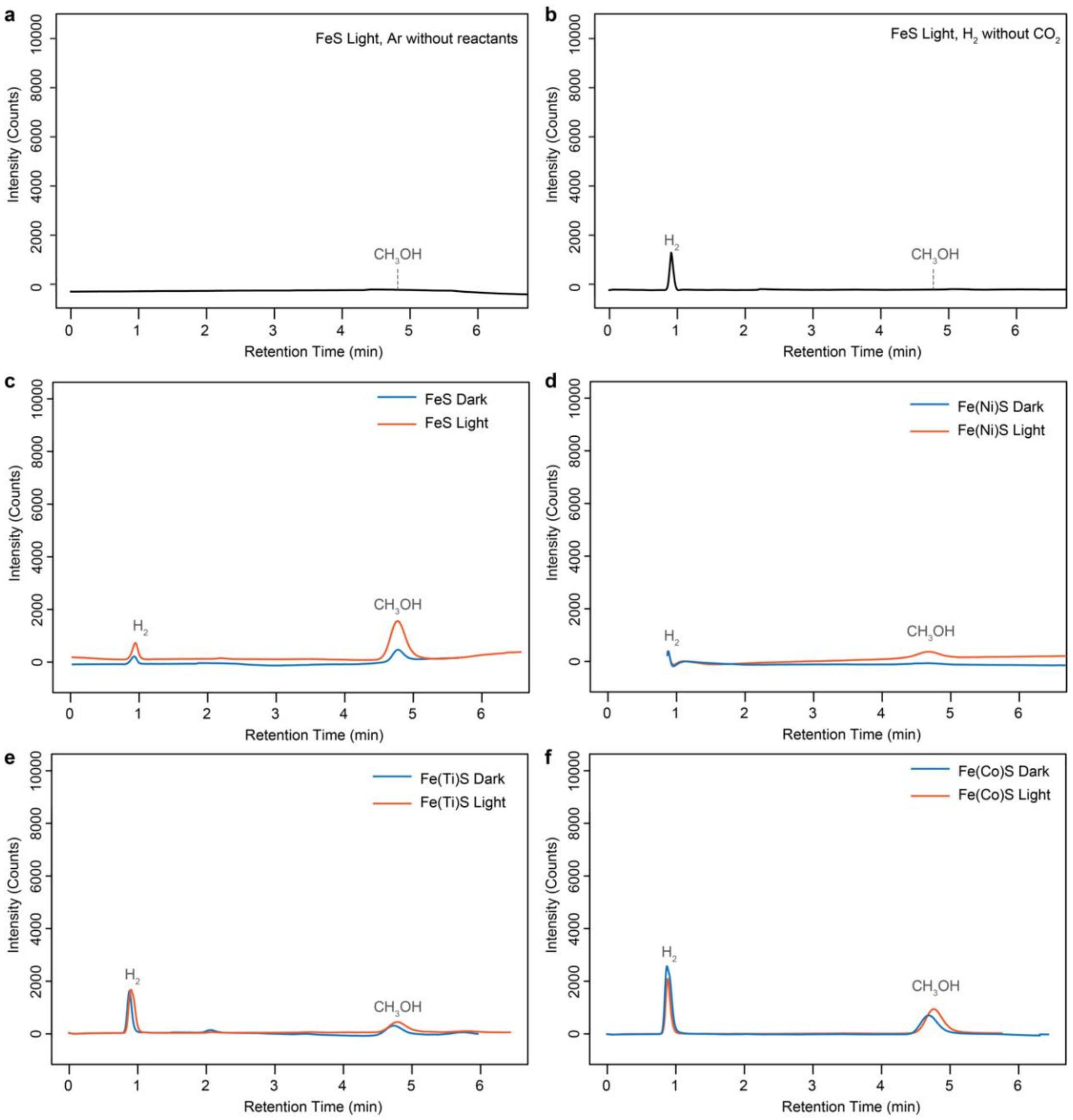

Figure S6. CH_3OH detection via GC-FID in pure, Ni-doped, Ti-doped, and Co-doped FeS experiments (doped at 10%, 120 °C under ambient pressure). **a** GC chromatogram at 120 °C without reactant over FeS. **b** GC chromatogram at 120 °C with H_2 as only reactant gas over FeS. **c–f** Representative GC chromatograms of CH_3OH detection over FeS, Ni-doped FeS, Ti-doped FeS, and Co-doped FeS at 120 °C under light and dark condition. To further affirm the absence of organic contamination, the pure FeS was subjected to a stringent thermal treatment at 120 °C under an argon (Ar) atmosphere and ambient pressure, yielding no detectable by-products. Furthermore, CH_3OH was not detected at 120 °C involving pure FeS and H_2. See Figure S7 for GC-FID chromatograms of H_2, H_2O, CO_2, and CH_3OH standards.

Table S2. Summary of examined iron sulfide catalysts and experimental conditions.

Catalyst	Reactants	Temperature (°C)	Light irradiation	Mass specific activity (μmol CH_3OH g^{-1} min^{-1})	Error bar	Mass specific activity (μmol CH_4 g^{-1} min^{-1})	Error bar
FeS	$3H_2 + CO_2$	120	None	0.6	0.1	-	
FeS	$3H_2 + CO_2$	110	None	0.5	0.0	-	
FeS	$3H_2 + CO_2$	100	None	0.4	0.0	-	
FeS	$3H_2 + CO_2$	90	None	0.4	0.0	-	
FeS	$3H_2 + CO_2$	80	None	0.2	0.0	-	
FeS	$3H_2 + CO_2$	120	UV–vis	2.6	0.2	-	
FeS	$3H_2 + CO_2$	110	UV–vis	1.6	0.2	-	
FeS	$3H_2 + CO_2$	100	UV–vis	1.4	0.1	-	
FeS	$3H_2 + CO_2$	90	UV–vis	1.1	0.1	-	
FeS	$3H_2 + CO_2$	80	UV–vis	0.9	0.1	-	
Fe(Ti)S	$3H_2 + CO_2$	120	None	0.3	0.0	-	
Fe(Ti)S	$3H_2 + CO_2$	120	UV–vis	0.4	0.0	-	
Fe(Co)S	$3H_2 + CO_2$	120	None	0.9	0.0	-	
Fe(Co)S	$3H_2 + CO_2$	120	UV–vis	1.0	0.1	-	
Fe(Ni)S	$3H_2 + CO_2$	120	None	0.1	0.0		
Fe(Ni)S	$3H_2 + CO_2$	120	UV–vis	0.4	0.0	0.2	0.0
Fe(Mn)S	$3H_2 + CO_2$	120	None	2.8	0.2	-	
Fe(Mn)S	$3H_2 + CO_2$	110	None	2.4	0.1	-	
Fe(Mn)S	$3H_2 + CO_2$	100	None	1.9	0.1	-	
Fe(Mn)S	$3H_2 + CO_2$	90	None	1.6	0.1	-	
Fe(Mn)S	$3H_2 + CO_2$	80	None	1.4	0.0	-	
Fe(Mn)S	$3H_2 + CO_2$	120	UV–vis	3.9	0.1	-	
Fe(Mn)S	$3H_2 + CO_2$	110	UV–vis	3.4	0.1	-	
Fe(Mn)S	$3H_2 + CO_2$	100	UV–vis	2.9	0.1	-	
Fe(Mn)S	$3H_2 + CO_2$	90	UV–vis	2.7	0.1	-	
Fe(Mn)S	$3H_2 + CO_2$	80	UV–vis	2.2	0.1	-	
Fe(Mn)S	$3H_2 + CO_2 + H_2O$	120	None	0.9	0.2	-	
Fe(Mn)S	$3H_2 + CO_2 + H_2O$	110	None	0.5	0.1	-	
Fe(Mn)S	$3H_2 + CO_2 + H_2O$	100	None	0.4	0.0	-	
Fe(Mn)S	$3H_2 + CO_2 + H_2O$	90	None	0.3	0.0	-	
Fe(Mn)S	$3H_2 + CO_2 + H_2O$	80	None	0.3	0.0	-	
Fe(Mn)S	$3H_2 + CO_2 + H_2O$	120	UV–vis	5.6	0.8	-	
Fe(Mn)S	$3H_2 + CO_2 + H_2O$	110	UV–vis	3.6	0.4	-	
Fe(Mn)S	$3H_2 + CO_2 + H_2O$	100	UV–vis	1.3	0.0	-	
Fe(Mn)S	$3H_2 + CO_2 + H_2O$	90	UV–vis	1.1	0.1	-	
Fe(Mn)S	$3H_2 + CO_2 + H_2O$	80	UV–vis	0.8	0.1	-	
Fe(Mn)S	$3H_2 + CO_2 + H_2O$	120	UV-enhanced	1.6	0.3	-	
Fe(Mn)S	$3H_2 + CO_2 + H_2O$	110	UV-enhanced	0.8	0.1	-	
Fe(Mn)S	$3H_2 + CO_2 + H_2O$	100	UV-enhanced	0.5	0.0	-	

4. H_2-Dependent CO_2 Reduction by Mn-Doped FeS Under Simulated Hot Spring Conditions

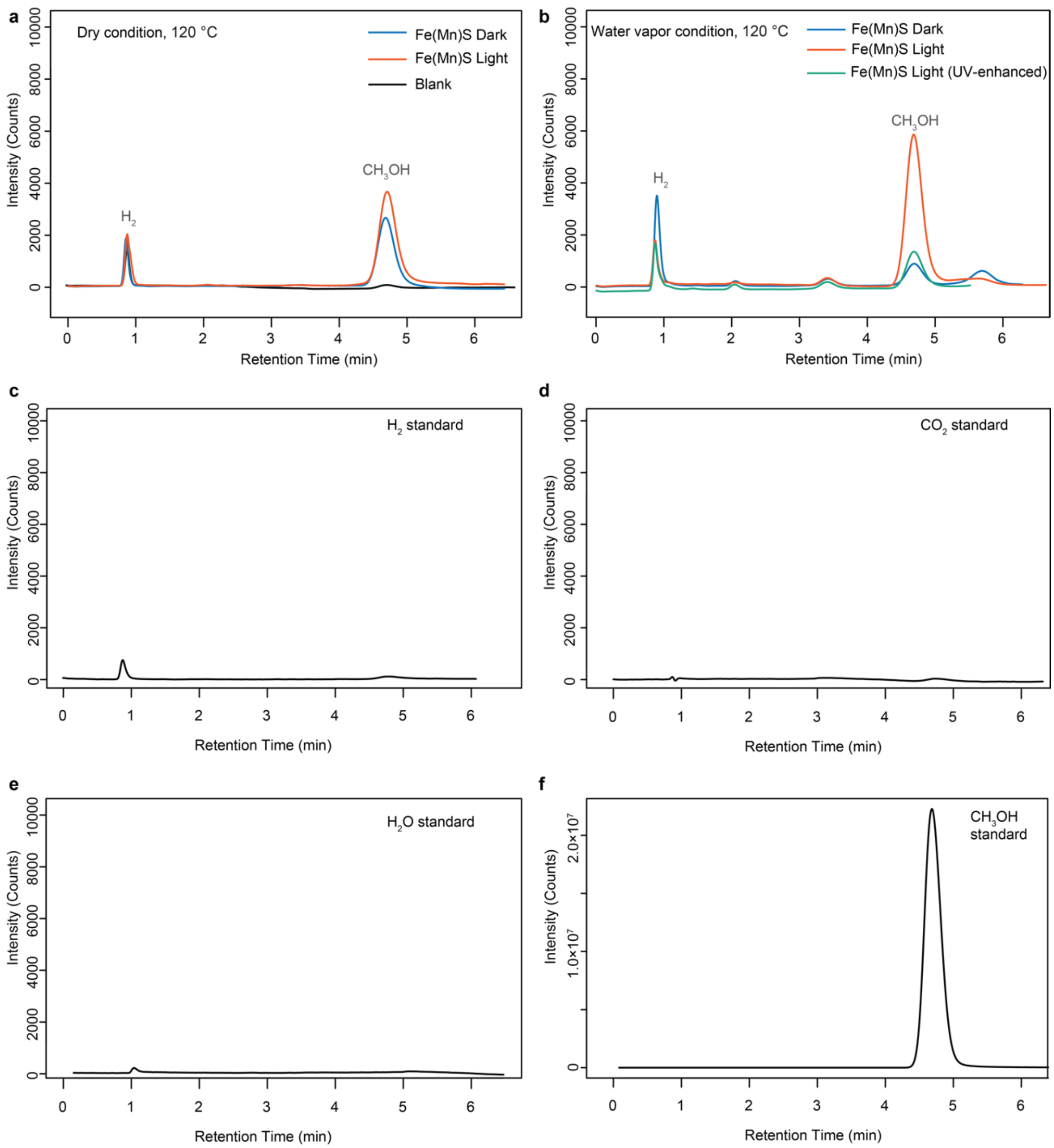

Figure S7. CH_3OH detection via GC-FID in Mn-doped FeS experiments (doped at 10%, 120 °C under ambient pressure). **a** CO_2/H_2 reaction. The blank control was conducted at room temperature without exposure to heat and light. **b** $CO_2/H_2/H_2O$ reaction. These results indicate the formation of CH_3OH at all tested conditions. Additional smaller peaks were detected, but their identities remain unclear as they have not been elucidated through the spiking of formic acid, formaldehyde, carbon monoxide, acetaldehyde, methane, or ethanol standards. **c–f** GC-FID chromatograms of H_2, CO_2, H_2O, and CH_3OH standards, respectively.

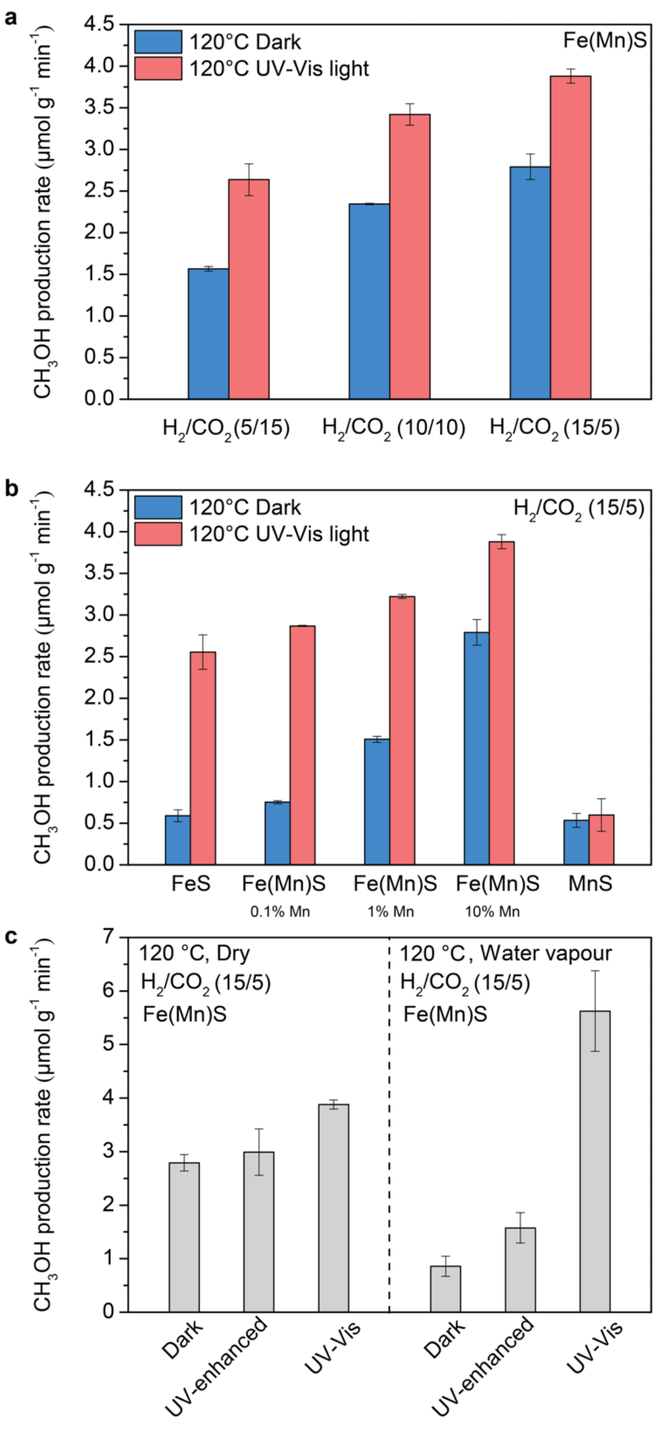

Figure S8. CO_2 reduction with H_2 catalyzed by Mn-doped FeS at 120 °C. **a** Impact of differing H_2/CO_2 ratios (5/15, 10/10, and 15/5) on Fe(Mn)S catalysis under dark and UV−visible light conditions. **b** Influence of various Mn doping ratios at 0.1%, 1%, and 10% on FeS catalysis in both dark and UV−visible light conditions. **c** Effect of dry and water vapor conditions on Fe(Mn)S catalysis under dark and light conditions with different light sources (UV-enhanced or UV−visible light). Unless specifically stated otherwise, all Fe(Mn)S refers to Mn-doped FeS at 10%.

5. Parameters Used in the DFT Calculations

Table S3. Parameters used in the DFT calculations.

Adsorbed species on FeS	∫C_vdT	EZPE	(300K) TS	Adsorbed species on Mn-doped FeS	∫C_vdT	EZPE	(300K) TS
H_2O(g)	0.104	0.573	0.584	H_2O(g)	0.104	0.573	0.584
H_2(g)	0.091	0.284	0.403	H_2(g)	0.091	0.284	0.403
CO_2(g)	0.091	0.308	0.652	CO_2(g)	0.091	0.308	0.652
CH_3OH(g)	0.123	1.344	0.756	CH_3OH(g)	0.123	1.344	0.756
*CO_2, H_2O	0.103	0.888	0.219	*CO_2, H_2O	0.103	0.886	0.221
*CO_2H, *H	0.105	0.776	0.203	*CO_2H, *H	0.111	0.769	0.222
*CO, H_2O	0.140	0.804	0.300	*CO, H_2O	0.140	0.800	0.296
*CO, H_2	0.103	0.605	0.204	*CO, H_2	0.097	0.606	0.190
*COH, *H	0.087	0.630	0.183	*COH, *H	0.083	0.634	0.157
*CHO, *H	0.092	0.549	0.191	*CHO, *H	0.115	0.604	0.261
*CHOH	0.072	0.774	0.151	*CHOH	0.076	0.763	0.159
*CHOH, H_2	0.099	1.169	0.184	*CHOH, H_2	0.108	1.153	0.203
*CH_2OH+*H	0.118	1.182	0.244	*CH_2OH+*H	0.118	1.182	0.244
*CH_3OH	0.122	1.365	0.267	*CH_3OH	0.094	1.361	0.193

6. SI Methods

Surface area analysis on FeS

The specific surface area of the samples was determined using nitrogen adsorption/desorption isotherms, conducted on a BELSORP MINI X adsorption analyzer at 77 K. Before analysis, the samples underwent pre-degassing under vacuum to ensure the removal of any adsorbed gases or moisture that could affect the measurement accuracy. The Brunauer-Emmett-Teller (BET) method was subsequently applied to calculate the specific surface area based on the adsorption data.

X-ray diffraction

For the X-ray diffraction (XRD) analysis, the sample preparation was conducted within an anaerobic chamber under a gas atmosphere of $N_2/CO_2/H_2$ = 85/10/5. The sample was securely placed in an airtight specimen holder designed by Bruker. Once preparation was complete, the holder was carefully removed from the anaerobic chamber and subsequently analyzed under ambient air conditions. Specifically, the XRD measurements were performed using a Bruker D2 Phaser, second generation, equipped with a Cu K-alpha radiation source (wavelength = 1.54184 Å). The diffraction data were collected with a step interval of 0.02° in 2 theta, and a counting time of 0.5 seconds per step. The scan range was set from 10° to 90° in 2 theta. The instrument operated at an accelerating voltage of 30 kV and a current of 10 mA.

For determining the crystalline size of the nanoparticles, the Debye-Scherrer equation was employed: $D = K\lambda/\beta\cos\theta$. In this equation, 'D' represents the crystalline size of the nanoparticles, 'K' is the Scherrer constant valued at 0.98, 'λ' is the wavelength of the X-ray source used in XRD, set at 1.54 Å, and 'β' is the full width at half maximum (FWHM) of the peak observed in the XRD pattern. This calculation aids in estimating the average size of the crystalline domains within the nanoparticles.
